# Coagulation Signaling through PAR1 as a Therapeutic Target in Pancreatic Ductal Adenocarcinoma

**DOI:** 10.3390/ijms22105138

**Published:** 2021-05-12

**Authors:** Aditi Kothari, Matthew J. Flick

**Affiliations:** Lineberger Comprehensive Cancer Center, and the UNC Blood Research Center, Department of Pathology and Laboratory Medicine, University of North Carolina at Chapel Hill, Chapel Hill, NC 27599, USA; aditik@email.unc.edu

**Keywords:** PDAC, thrombin, protease-activated receptor, antitumor immunity

## Abstract

Pancreatic ductal adenocarcinoma (PDAC) is a highly fatal disease with a 5-year survival rate of less than 10% following diagnosis. The aggressive and invasive properties of pancreatic cancer tumors coupled with poor diagnostic options contribute to the high mortality rate since most patients present with late-stage disease. Accordingly, PDAC is linked to the highest rate of cancer-associated venous thromboembolic disease of all solid tumor malignancies. However, in addition to promoting clot formation, recent studies suggest that the coagulation system in PDAC mediates a reciprocal relationship, whereby coagulation proteases and receptors promote PDAC tumor progression and dissemination. Here, upregulation of tissue factor (TF) by tumor cells can drive local generation of the central coagulation protease thrombin that promotes cell signaling activity through protease-activated receptors (PARs) expressed by both tumor cells and multiple stromal cell subsets. Moreover, the TF-thrombin-PAR1 signaling axis appears to be a major mechanism of cancer progression in general and PDAC in particular. Here, we summarize the current literature regarding the role of PAR1 in PDAC and review possibilities for pharmacologically targeting PAR1 as a PDAC therapeutic approach.

## 1. Introduction

Pancreatic cancer develops from unrestricted and abnormal growth of cells in the tissues of the pancreas. It is the ninth most common type of cancer in women and tenth most common type of cancer in men [1]. Pancreatic cancer originates from multiple cells with 93% of cases being exocrine adenocarcinomas and the remaining 7% being neuroendocrine type tumors. Being the fourth most deadliest cancer, pancreatic cancer accounts for 8% of all cancer deaths with an average 5 year survival rate of 9% [1]. It is predicted that pancreatic cancer will become the second most common cause of cancer deaths by 2030, [2] due to a combination of an increased pancreatic cancer incidence rate, routinely late detection of pancreatic tumors, and ineffective current treatment options. Only 10–15% of pancreatic cancers can be detected at a stage where the cancer has not spread to any nearby organs and can be surgically removed. The 5-year survival rate at this stage is 37% [3]. A small number of these tumors may not be surgically removed initially, but upon treatment with chemotherapy or radiation therapy, it is possible to sufficiently reduce the tumor and then surgically remove it. Around 35–40% of pancreatic cancers are diagnosed at a stage where they have migrated to nearby organs and veins, and surgically removing them is not possible [4]. Metastatic tumors account for 45–50% of cancers that are diagnosed when they spread to other organs like liver, lungs, or parts of the abdomen [4]. It is imperative to fill knowledge gaps related to early detection of premalignant conditions, comorbidities, mechanisms of pathogenesis, and pathways rendering pancreatic cancer refractive to therapy in order to improve surgical and medical management of this deadly disease.

Pancreatic cancer is predominantly identified in the elderly, and it is extremely rare for it to be observed in individuals under 30 years of age [5]. Cases are more prominent in developed countries, which suggests that lifestyle choices may contribute to PDAC development [6]. One study aimed to draw a correlation between global temporal patterns and socioeconomic development showed that around 55% of total incidences and 56% of mortality due to pancreatic cancer occurred in more developed areas in the world [6]. It is also more common in men as compared to women [5]. However, a review of 15 studies ruled out the involvement of reproductive factors in women as a reason for the higher number [7]. A study by the Pancreatic Cancer Cohort Consortium reported that blood groups A, B, or AB were at a higher risk of developing pancreatic cancer as compared to people with blood group O [8]. Differences in host inflammatory state and glycosyltransferase specificity across different blood types were some of the proposed mechanisms supporting this finding [8]. Individuals with a family history of pancreatic cancer, obesity, and diabetes were also found to be more prone to pancreatic cancer, pointing to the fact that genetic susceptibility and family history do play a role in tumor development, the most common mutations cited being BRCA2 and PALB [9]. Chronic pancreatitis, a condition involving inflammation of the pancreas along with loss of acinar and islet cells, is also associated with an increased risk of pancreatic cancer [10]. Lifestyle choices like smoking and excess alcohol consumption are speculated to be involved [11]. Collectively, the overall risk of developing pancreatic cancer is linked to a combination of genetic and environmental factors.

The most common type of pancreatic cancer is pancreatic ductal adenocarcinoma (PDAC), a type of cancer that originates in cells of the exocrine portion of the pancreas. Acinar cells, which produce digestive enzymes and other products of the exocrine pancreas, and cells that line the exocrine ducts are the cells of origin for PDAC neoplasms. The acquisition of oncogenic mutations in ductal or acinar cells leads to cellular transformation and induction of PDAC. Precursor lesions termed pancreatic intraepithelial neoplasia (PanINs) represent the earliest histologically evident changes of PDAC [12]. PanINs are non-invasive, microscopic lesions, usually less than 0.5 cm in size. Low-grade PanINs typically have activating KRAS mutations along with telomere shortening, pointing to a pathway towards malignancy [13]. High-grade PanIN and PDAC also have loss of function p16, p53, CDNK27, and SMAD4 mutations. The frequency of KRAS mutations increases as the grade of tumor increases [14]. There have also been reports of irregularities in notch signaling and sonic hedgehog pathway as PDAC progresses that could play a role in the development of pancreatitis and exacerbate tumor progression [15].

The high mortality rates of PDAC patients highlights the failure of current treatment strategies. An overarching problem for therapy is the difficulty in identifying and diagnosing patients at early stages of the disease. Magnetic resonance imaging (MRI), magnetic resonance cholangiopancreatography (MRC) along with endoscopic ultrasound (EUS) are common methods of screening. Serum cancer antigen 19-9 (CA 19-9) is one biomarker approved by the US FDA, known to increase in 75–85% PDAC patients. However, CA 19-9 is typically used as a marker in recurring disease as opposed to screening asymptotic patients [16]. Although a number of surgical strategies are considered, pre-operative biliary drainage, anastomotic technique, minimal invasive surgery, and vascular resection are some surgical management techniques for PDAC [17]. Invasion and metastasis of the primary tumor to nearby and anatomically distant organs often renders the malignancy non-resectable by the time it is detected. Medical management employing various chemotherapeutic regimens is the standard of care for the majority of PDAC patients. Gemcitabine, FORLFIRINOX, irinotecan, oxaliplatin, and fluorouracil are some chemotherapeutics used alone or in combination [18]. These strategies have not shown to improve patient survival significantly. Neo-adjuvant therapy is given to patients with marginally resectable or early-stage tumor, in which case chemotherapy is provided before surgery [19]. The role of radiation in treatment is still not clear, with some data suggesting radiation in neo-adjuvant setting to be helpful [20]. Immunotherapy has opened new gates in the field of cancer biology. Yet, the effectiveness of immunotherapy in the context of PDAC has been limited to date due in large part to a potently immune-suppressive, therapy-resistant microenvironment in PDAC. The identification of pathways and mechanisms that render the PDAC microenvironment immune permissive and therapy sensitive offers a novel strategy for significantly enhancing current chemo- and immunotherapy strategies.

## 2. Thrombosis in Cancer

The association of thrombosis and cancer has been long recognized. Thrombotic disorders in cancer patients were first recorded in the 1860s when French physician Armand Trousseau noted an increased frequency of spontaneous coagulation due to ‘special crisis’ in their blood [21]. In the diagnosis of cancer, venous thromboembolic disease (VTE) is now observed as the first clinical manifestation, and the second leading cause of death due to cancer [22]. A correlation between thromboembolic events and poor prognosis of cancer has been drawn in context of multiple cancer types, showing that activation of blood coagulation leads to a more aggressive tumor phenotype. The median survival increases from 12% to 36% in patients that do not report thromboembolic events associated with cancer [23]. These findings may be due to associated thromboembolic events but could also be due to a more aggressive tumor. One report indicated that disseminated intravascular coagulation (DIC) leads to a poor prognosis in patients with solid tumors, irrespective of their association with thrombosis [24]. Collectively, these studies highlight the interrelationship between blood coagulation and cancer pathogenesis.

Pancreatic cancer is of particular interest for cancer-associated thrombosis as pancreatic cancer is the number one cancer-associated with VTE [25]. In pancreatic cancer, elevated plasma levels of fibrinogen, factor (F) VIII, and D-dimers have been seen along with reduced levels of protein C and antithrombin III [26]. Although the exact causes remain elusive, multiple mechanisms have been proposed for PDAC-driven thrombosis. Tissue factor, a transmembrane receptor, and the initiator of the coagulation cascade can drive aberrant coagulation activity in PDAC [27]. When pancreatic cells undergo a malignant transformation, an early transcriptional event is high level expression of TF by the newly formed tumor cell. TF is also expressed on the surface of stromal cells and subsets of infiltrating inflammatory cells. Tumor-derived TF can be released from the tumor in the form of TF+ microvesicles that drive clot formation and thrombosis once entering the circulation as has been suggested in patient studies and mouse models [28,29,30].

Altered fibrinolysis (i.e., process of blood clot dissolution) also may contribute to VTE in PDAC. Plasminogen activator inhibitor type 1 (PAI-1) is an inhibitor of fibrinolysis, whose levels are substantially upregulated in PDAC [31]. High levels of PAI-1 can inhibit plasminogen activation thereby promoting the persistence and possible expansion of clots that form. Further, it was recently shown that nude mice bearing PDAC tumors following orthotopic injection of the human PDAC cell line PANC-1 had increased levels of both active human and active mouse PAI-1 and decreased levels of plasmin activity [32]. Notably, mice bearing PANC-1 tumors exhibited impaired venous thrombus resolution 8 days after induction of thrombosis by complete ligation of the inferior vena cava compared with nontumor controls [33]. Whether alterations in other plasminogen activation system components influences VTE in PDAC remain to be established.

Inflammatory cytokines like interleukin-1 (IL-1), tumor necrosis factor-α (TNF-α), and VEGF are secreted by PDAC cells and may also promote hypercoagulability and thrombosis through at least two different mechanisms. First, inflammatory mediators can activate vascular endothelial cells (ECs). Activation of ECs can exacerbate thrombosis by multiple mechanisms including induction of TF, downregulating thrombomodulin (mediator of thrombin generation), promoting PAI-1 synthesis, and exposure of phosphatidylserine (PS) on the cell surface [34,35,36,37,38]. Furthermore, activated endothelium can support platelet accumulation and aggregation in pancreatic cancer via a thrombin-dependent pathway that contributes to the initial stages of hypercoagulation in these cells. Second, inflammation in PDAC can result in neutrophil activation and the formation of neutrophil extracellular traps (NETs) [39,40,41]. NETs have been strongly linked to thrombosis in a variety of contexts, including cancer [42,43]. Importantly, there may not be one unified mechanism mediating VTE in PDAC, but a constellation of pathological changes that ultimately result in a devastating vaso-occlusive event.

## 3. Coagulation Activity as a Modifier of the PDAC Disease Progression

Studies utilizing in vitro and in vivo genetic manipulations have identified clotting factors that play a reciprocal role in promoting tumor invasion, angiogenesis, and metastasis [44]. Histological analyses of cancer tissues have found fibrin and platelet aggregates around the tumor cells, pointing to local activation of coagulation. Aberrations in blood clotting are also observed in more than 60% of cancer patients, regardless of observed thrombosis [45]. Increased TF has been associated with both pro-coagulant activity and aggressiveness of the tumor [46], suggesting a possible functional link between altered coagulation and PDAC progression. Several of the known PDAC tumorigenic mutations, including mutations in KRAS, EGFRvIII (epidermal growth factor receptor variant III), HER-2 (human epidermal growth factor receptor 2), as well loss of function mutations of p53 and PTEN (phosphate and tensin homolog) each can drive TF overexpression. Cancer cells have an elevated level of PS on the surface of their outer membrane [47]. High levels of PS and TF on the outer membrane can create a nidus for local coagulation factor activity, culminating in thrombin activation and the subsequent fibrin deposit in the extravascular space of the tumor microenvironment (TME). TF expression in pancreatic cancer tumors has been associated with tumor development and metastasis [48]. Tissue factor expression also increases with histologic grade in different cancer types, including pancreatic cancer [28]. For PDAC, TF was shown to be a powerful determinant of primary tumor growth and metastatic potential in that genetic elimination of TF by gene editing in C57Bl/6-derived mouse PDAC ‘KPC’ cells (i.e., Kras^G12D^; p53^R172H^) resulted in significantly diminished tumor growth and experimental metastasis when cells transplanted back into C57Bl/6 immune competent mice [49].

Alternatively spliced tissue factor (asTF), a soluble isoform of the full-length tissue factor (flTF), is a minimally coagulant signaling molecule that activates various signaling pathways, including PI3K/AKT, MAPK, and FAK pathways by binding to α6β1 and αvβ1 non-proteolytically [50]. Interaction of asTF with β1 integrin increases the expression of cell adhesion molecules like VCAM-1, ICAM-1, and E-selectin [51]. The number of tumor-associated monocytes/macrophages (TAMs) increases as the levels of asTF rises, suggesting that asTF might be contributing to monocyte/macrophage recruitment. Since TAMs play a role in tumor progression and resistance to chemotherapy, there is a possibility that asTF might also play a role in the pathway. Expression of asTF is observed in early neoplastic PDAC lesion (PanIN) through advanced stages. It has been shown that asTF interacts with PDAC cells to promote tumor cell growth, proliferation, and spread as well as monocyte accumulation in the tumor microenvironment. Accordingly, asTF is now being considered as a therapeutic target for PDAC [52]. One study documented the effects of delayed induction of asTF in PDAC and the use of asTF antibody to treat PDAC growth and metastasis [53]. According to this study, asTF binds to β1 integrins on the surface of PDAC cells, thereby promoting tumor growth, metastasis and immune cell infiltration in the stroma. Testing the therapeutic efficacy of targeting asTF in PDAC, this study reports the use of asTF antibody to reduce PDAC tumor progression. Tumor-derived asTF induced PDAC tumor growth and metastasis in the initial as well as later stages of the disease. Considering the role of asTF in PDAC spread in host cells, this study points to asTF as a potent target in PDAC.

## 4. Protease-Activated Receptor-1 Signaling in Cancer Progression

The canonical function of native TF is the generation of the central coagulation protease thrombin. Thrombin is essential for blood clotting as it mediates conversion of the soluble glycoprotein fibrinogen to fibrin, which spontaneously polymerizes to form the structural component of the blood clot. However, thrombin can mediate cell signaling events through activation of a class of receptors called protease-activated receptors (PARs). PARs are a family of seven transmembrane, G-protein-coupled receptors. Four types of PARs are present in mammals, of which PAR1, 3 and 4 are activated by thrombin, while PAR2 can be activated by FVIIa and FXa but not thrombin [54]. PARs are usually overexpressed in cancer, and reports draw a correlation between PAR expression and aggressive tumor phenotype [55].

PAR1 signaling has been studied in a number of tumor models and results have revealed a complicated picture suggesting that the contribution of PAR1 may be tumor type specific. One study utilized two spontaneously developing murine intestinal adenomas and reported a more aggressive tumor progression after elimination of PAR1 [56]. They highlight the role of PAR1 in promoting apoptosis of transformed cells in vivo. This mechanism limited tumor growth potential that occurs at a relatively early stage in mice [56]. Interestingly, another study revealed that in the setting of breast cancer, PAR2 and not PAR1 might be responsible for tumor progression [57]. Their results show no effect of PAR1 on tumor growth, whereas PAR2 was shown to be involved in an ‘angiogenic switch’, where the cells without PAR2 were in a state of dormancy by blocking proangiogenic signaling in the tumor model [57]. A few other studies have pointed out that PAR1 overexpression alone may not lead to aggressive tumor phenotypes, and may be dependent on other pathways like Rho-mediated signaling [58] and Wnt-mediated β-catenin stabilization [59]. All this points to a context-dependent role of PAR1 in tumorigenesis.

**Figure 1:**
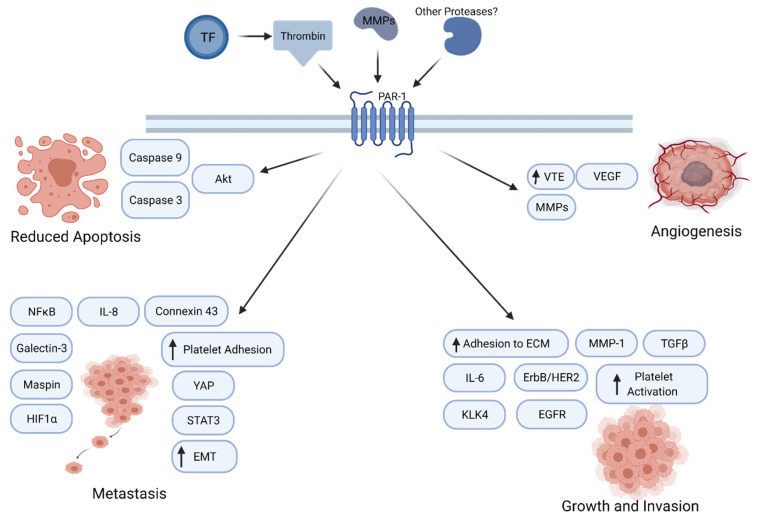
PAR1 signaling in cancer progression. PAR1 promotes tumor growth, angiogenesis, invasion, and metastasis through multiple molecular mechanisms.

A number of studies indicate that PAR1 enhances tumor growth through a variety of mechanisms (Figure 1). In a rat model of benign tumor, PAR-mediated silencing of pro-apoptotic genes leads to tumor growth and invasion [60]. PAR1 also interacts with epidermal growth factor receptor (EGFR) or ErbB/HER2 to regulate calcium pathway in cancer cells [61]. PAR1 signaling augments the Galectin-3 and Hippo-YAP pathways to enhance tumor cell motility and promote tumorigenesis [62]. PAR1 was also observed to activate the Akt pathway, leading to reduced expression of caspase 3 and caspase 9, thereby leading to a diminution of apoptosis. A paracrine mechanism also shows production of KLK4 which, in turn, releases IL-6 in a PAR1-regulated manner, which then stimulates cancer cells to grow and proliferate [63]. Thrombin-PAR1 signaling has been associated with increased angiogenesis via VEGF production and release of matrix metalloproteinases (MMPs) [64]. PAR1 and PAR-2 work together to promote tumor growth in breast cancer.

PAR1 has also been shown to play a role in tumor invasion and metastasis in multiple tumor cells (Figure 1) [65,66,67]. PAR1 increases adhesion to extracellular matrix, thereby increasing invasiveness. One study showed that NF-κB-dependent activation of PAR1 leads to tumor growth and invasion [68,69]. One study reported that PAR1 regulated the levels of tumor suppressors Maspin and connexin 43, thereby inducing a metastatic phenotype [70]. Inhibition of PAR1 or thrombin lead to a decrease in levels of MMP-2, IL-8 and VEGF expression levels, leading to reduced metastasis [71]. PAR1 signaling has also been implicated in epithelial to mesenchymal transition (EMT) in multiple cancer types. Thrombin-PAR1 signaling increases platelet adhesion, and thrombin-induced HIF1α increases mRNA levels of torsion, which can lead to EMT and increase tumor metastasis [72]. Additionally, in gastric cancer cells, thrombin-activated PAR1 leads to an increased EMT. In colon cancer, PAR1 activation induces HIF-1α levels to promote metastasis [73]. In renal cell carcinoma, PAR1 leads to metastasis and survival, along with more STAT3-dependent activation of the receptor [74]. In non-small cell lung cancer (NSCLC), SiRNA-mediated knockdown of PAR1 inhibited lung adenocarcinoma growth and invasion significantly, whereas PAR1 expression was increased by TGFβ [75,76]. Collectively, these findings implicate PAR1 function at multiple stages of cancer progression.

## 5. Thrombin/PAR1 Signaling in PDAC

**Figure 2:**
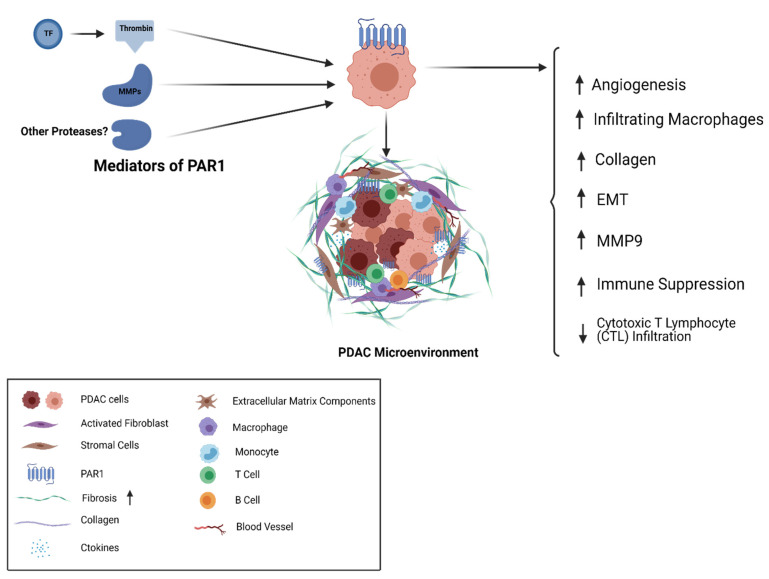
PAR1 in PDAC; TF-Thrombin and MMPs are agonists of the cell surface receptor PAR1 that is expressed on the surface of PDAC tumor cells and stromal cells. PAR1 activity shapes multiple elements of the cancer cell and stromal cell repertoire as well as the desmoplastic matrix associated with PDAC.

A number of PAR1-mediated mechanisms have been implicated for PDAC (Figure 2). For example, pancreatic cancer is characterized by a desmoplastic stroma with substantial fibrosis around tumor cells. Secretion of collagen and other extracellular matrix components takes place by activated fibroblasts in the tumor microenvironment. The stroma plays a very unique role, not just as a mechanical and physical barrier but also in tumor progression, metastasis, and drug resistance [77]. Considering the role of PAR1 in other tumor types, and the distinctive pancreatic tumor microenvironment, it is possible that PAR1 is driving the desmoplastic reaction and promoting tumor growth and resistance to treatment.

One group investigating the role of PAR1 in PDAC reported a dual role for PAR1 in the stroma and tumor. Stromal cells expressing PAR1 seemed to promote an aggressive tumor phenotype [78], whereas tumor cells without PAR1 increased tumor growth [79]. Their study suggested a compartmentalized approach to treatment of PDAC, where only stromal cells should be targeted [79]. However, it is worthwhile to note that this study utilizing shRNA-mediated knockdown of PAR1 in PDAC cells proceeded by orthotopic implantation of these cells failed to show control experiments with PAR1 ‘addback’ followed by implantation of the rescued cells in the mouse model to confirm the specificity of the knockdown approach.

PAR1 expression has been observed in human PDAC tumors and metastatic sites. This expression coincided with the expression of stromal markers along with evidence of desmoplasia. Some reports suggest that PAR1 plays a role in blood vessel formation and angiogenesis, while angiogenesis is a known hallmark for cancer and tumor growth [80,81,82]. Interestingly, PAR1-deficient mice showed angiogenic changes in a PDAC tumor model while wildtype tumors had a higher expression of CD31 [78]. Gemcitabine, a widely used chemotherapeutic in pancreatic cancer, showed complete tumor reduction in PAR1-deficient mice, while wildtype tumors showed two-fold tumor reduction [78]. The desmoplastic stroma in PDAC plays a significant role in drug resistance, [83] and cancer-associated fibroblasts are known to exacerbate tumor growth [84]. However, markers for fibrosis such as alpha-smooth muscle actin (aSMA) levels and collagen (in immunohistochemical staining with trichrome stain) were not different between the two tumor models. PAR1-proficient tumors also had a higher number of infiltrating macrophages, as compared to PAR1-deficient mice, and these tumors also showed reduced gemcitabine sensitivity [78]. Tumor-associated macrophages have previously been reported to be associated with tumor growth and chemoresistance [85]. Collectively, these findings may point to a role of PAR1 on endothelial cells in promoting tumor angiogenesis, and on macrophages to induce chemoresistance [86,87]. The impact on macrophages could be linked to M2-like macrophage induction of EMT transition [88] and blocking macrophages has previously been reported to reduce metastasis [89].

One study attempted to unravel the relationship between PAR1, macrophages, and PDAC tumor cells. In this report, tumor tissue showed an increased level of macrophage marker CD68 and PAR1 marker F2R. Interestingly, immunohistochemical analysis did not show expression of PAR1 on CD68 and CD163 (marker for tumor-associated macrophages), pointing to these macrophages being PAR1 negative [90]. PANC-1 pancreatic cancer cells grown in conditioned media from PMA-induced THP1 monocytes showed fibroblast-like morphological changes. These changes seemed to be ablated by the PAR1 inhibitor vorapaxar and shRNA-mediated silencing of PAR1 on PANC-1 cells. PANC1 cells grown in conditioned media also showed a decrease in the expression of E-cadherin (CDH1) and an increase in expression of zinc finger E-box binding homeobox 1 (ZEB1) and vimentin (VIM), pointing to a PAR1/macrophage-induced mesenchymal tumor state in PDAC [90]. MMP9, previously suggested as a PAR1 cleaving/activating protease, showed a significantly higher expression in macrophages as compared to other proteases like granzyme B, proteinase K, and kallikrein 4. The N-terminal tethered ligand of PAR1 was also shown to have three potential MMP9 cleavage sites [91]. Interestingly, this site lies close to the thrombin cleavage site and showed the most robust proteolytic cleavage for the P1 position at Serine 42 [90]. It was further demonstrated that PAR1 activation by MMP9 leads to mesenchymal differentiation [90]. In accordance with the previously explored ability of mesenchymal transition to confer drug resistance, [92] the MMP9-PAR1 axis can facilitate cancer cell escape of macrophage-dependent cell death [90]. ZEB1-silenced PANC1 cells with blocked ability to undergo EMT also showed poor viability as compared to cells that were able to undergo mesenchymal transition. Further targeting MMP9-PAR1 axis in ZEB1 silenced cells did not exacerbate the cell death, suggesting this mechanism as a major mediator of cell death. Hence, PAR1-mediated mesenchymal transition may be one way by which PDAC cells protect themselves from macrophage-induced cytotoxicity [90]. These experiments point to a macrophage/PAR1 cross talk contributing to poor prognosis in PDAC.

Another recent study shows that CD8+ T cells play a crucial role in eliminating PDAC tumor cells in vivo by showing that PAR1 KO tumor formation was only seen in immune-compromised mouse and not in immunocompetent mice. By genetically modifying levels of pro-coagulant proteins in their mouse model, it was shown that tumor cell-derived TF, circulating prothrombin, and PAR1 are key mediators of PDAC progression. Metastatic assays performed on mice treated with shPAR1 and shTF KPC2 cells showed reduced metastatic potential in these tumors as compared to shControl cell tumors [93]. One mechanism of PAR1-/thrombin-mediated growth of PDAC is suppression of antitumor immunity in the tumor microenvironment [93]. A follow-up study investigating the cellular and molecular PAR1-mediated changes in the PDAC tumor microenvironment highlighted the significance of PAR1 in driving the antitumor response. Here, loss of PAR1 causes increased antitumor efficacy, making PDAC cells more prone to immune-mediated therapies. Genes like *Ptgs2* and *Csf2* that are downstream of thrombin-PAR1 immune response pathway, were also shown to be playing a role in PDAC tumorigenesis. Their absence alone, in the presence of PAR1 could restore tumor growth. Using syngeneic graft models, it was shown that PAR1 KO increased cytotoxic T lymphocyte infiltration (CTL) and decreased tumor-associated macrophages in the tumor microenvironment. PAR1-expressing and PAR1 KO tumor cells were injected together in immunocompetent mice. Interestingly, this resulted in preferential elimination of PAR1-KO cells from growing tumors, which suggested that PAR1-dependent immune evasion might not depend on CTL exclusion. No change in the expression of immune checkpoint proteins and major histocompatibility complex-I cell surface expression was observed that could be attributed as PAR1 dependent [94]. These findings highlighted the concept that PAR1-driven tumor growth in PDAC can be mediated by cross talk between coagulation and immune system components leading to tumor immune evasion.

PAR1 agonists and activators appear to contribute to PDAC growth and spread. Alternative means of PAR1 activation may include biased signaling through activated protein C (APC)-mediated PAR1 cleavage. APC-PAR1 pathway has downstream targets that are distinct from the ones in the thrombin/PAR1 signaling [95]. Hence, this pathway may also contribute to unique effects. Another PAR1 agonist is plasmin. One study in plasminogen-deficient mice showed reduced tumor growth as compared to mice with plasminogen. Further research is needed to define the precise mechanisms of action for distinct PAR1 activators in PDAC [94].

## 6. Targeting PAR1 as a Therapeutic Approach in PDAC

Given that PAR1 may be activated by multiple agonists, drug development of a selective inhibitor can be challenging. Further, binding of orthosteric inhibitors for PARs should be irreversible in order to compete with the tethered ligand. Cytoprotective signaling could be hampered due to the biased nature of pathways that come downstream of PAR1. All PAR1-targeted proteases (e.g., activated protein C (APC)), have alternate substrates, which may lead to off-target effects. The distribution of PAR1 varies across different tissues and cell types in the body of different species. Mice, rats, dogs and rabbits express PAR3 and PAR4 on their platelets, whereas humans express PAR1 and PAR4 on platelets [96]. This creates both limitations for the use of animal models in developing these therapeutics and a risk for targeting PAR1 in PDAC without influencing platelet-mediated hemostasis. However, various agents, including peptide-based agents, small molecules and therapeutic proteases are being developed to understand PAR1 signaling. These agents are designed to either inhibit PAR1 prothrombotic, proinflammatory, profibrotic, and tumor-promoting activities or activate PAR1-mediated cytoprotective pathway.

Immune checkpoint therapy for treatment of PDAC has not been successful (e.g., CTLA-4, PD-1) [97]. Immune checkpoint monotherapy in combination with vaccines or other chemotherapeutic drugs could be a strategy to tackle the failure of using immunotherapy alone in these tumors [98,99,100]. In regards to vaccine therapy in pancreatic cancer, one example is GVAX, which aids in secretion of granulocyte macrophage colony-stimulating factor (GM-CSF) and carries out an anti-tumor response [101]. Vaccines targeting specific oncoproteins like KRAS, MUC1, and VEGF-R are also being tested alone or in combination with GVAX [100]. CAR-T therapy targeting mesothelin, (an antigen overexpressed in PDAC), anti-CEA, and anti-CD33 are being tested and show good tolerance; however, these have failed to produce a good anti-tumor response [102,103,104,105]. Immune-modulating agents that target the dense stroma found in PDAC are being tested in combination with gemcitabine. Anti-CD40 agonist is one such agent being utilized that perturbs the stroma [106]. The working concept being that by ‘loosening’ the stroma anticancer agents will more effectively reach their target and eliminate tumor cells.

The various studies highlighting the role of PAR1 in mediating immune suppression point to PAR1 as a primary reason why PDAC has poor prognosis and immunogenicity. Considering that one of the early transcriptional changes that occurs downstream of driver mutations in PDAC is the increased expression of TF and PAR1 in the tumor cells/stromal cells, it follows these changes that confer immune evasion and tumor progression from the earliest stages of PDAC. PAR1 has been known to play a significant role in mediating immune response in inflammatory diseases and viral infections as well [107,108]. The immune suppressive nature of PAR1 makes PAR1 inhibitors good candidates for combination therapy with immune checkpoint drugs. CD8 T cells were seen as the key players in mediating an antitumor response in the absence of PAR1 [93]. This hints to other mechanisms beyond PAR1 that may drive an immune response. Further studies are needed to confirm the role of key players downstream of PAR1 that can be linked to the immune system. Given the preliminary data in PDAC and more established models in other cancer types, targeting PAR1 could prove to be an effective therapeutic strategy.

Vorapaxar is the first PAR1 inhibitor approved for clinical use [109]. Its main indication includes a reduction in thrombotic cardiovascular events in patients with previous indications of myocardial infarction and peripheral artery disease, owing to PAR1 inhibition suppressing platelet activation. Studies have reported that it is also effective against peripheral arterial disease, acute coronary syndrome, and pulmonary hypertension, among others. It is worthy to note that its use in cancer therapy is still largely untested. It has been shown to inhibit epithelial ovarian cancer progression, the most common side effect being bleeding [110]. Atopaxar is another PAR1 inhibitor that works via reducing platelet activation. It is currently under phase 2 trials, with the latest studies not very effective in preventing cure bleeding [111,112]. Therefore, while more research and development is underway, additional preclinical and clinical studies are required to determine the efficacy of PAR1-targeted therapeutics in suppressing PDAC disease progression. Given the documented tumor heterogeneity in PDAC [113] developing effective combination therapies would require characterizing patient samples. Therefore, characterizing tumors would help identify individual distinctions, which can then be targeted with combinatory approaches. Even though complete tumor resection might not be achieved, reduction in tumor size, control of metastasis, and limiting tumor recurrence might still add to patient survival.

## 7. Summary

The poor prognosis for pancreatic cancer patients makes it imperative to find new therapeutic targets and candidate molecules that inhibit tumor growth, invasion, and metastasis in PDAC. Such drugs might open new doors in clinical cancer treatment. To improve the PDAC treatment, more knowledge is needed on the mechanisms that promote PDAC growth. Considering the multiple deleterious effects of PAR1 in PDAC, PAR1 stands as an attractive target for therapy.

## Data Availability

Not Applicable.
